# Different diversification histories in tropical and temperate lineages in the ascomycete subfamily Protoparmelioideae (Parmeliaceae)

**DOI:** 10.3897/mycokeys.36.22548

**Published:** 2018-07-02

**Authors:** Garima Singh, Francesco Dal Grande, Jan Schnitzler, Markus Pfenninger, Imke Schmitt

**Affiliations:** 1 Senckenberg Biodiversity and Climate Research Centre (SBiK-F), Frankfurt am Main, Germany; 2 Department of Molecular Evolution and Plant Systematics, Institute of Biology, Leipzig University, Germany; 3 Department of Biological Sciences, Institute of Ecology, Evolution and Diversity, Goethe Universität Frankfurt am Main, Germany

**Keywords:** Diversification pattern, dating, extra-tropical, mountain uplifts, ancestral state reconstruction, substrate, habitat, parallel evolution, lichenised fungi

## Abstract

**Background**: Environment and geographic processes affect species’ distributions as well as evolutionary processes, such as clade diversification. Estimating the time of origin and diversification of organisms helps us understand how climate fluctuations in the past might have influenced the diversification and present distribution of species. Complementing divergence dating with character evolution could indicate how key innovations have facilitated the diversification of species.

**Methods**: We estimated the divergence times within the newly recognised subfamily Protoparmelioideae (Ascomycota) using a multilocus dataset to assess the temporal context of diversification events. We reconstructed ancestral habitats and substrate using a species tree generated in *Beast.

**Results**: We found that the diversification in Protoparmelioideae occurred during the Miocene and that the diversification events in the tropical clade *Maronina* predate those of the extratropical *Protoparmelia*. Character reconstructions suggest that the ancestor of Protoparmelioideae was most probably a rock-dwelling lichen inhabiting temperate environments.

**Conclusions**: Major diversification within the subtropical/tropical genus *Maronina* occurred between the Paleocene and Miocene whereas the diversifications within the montane, arctic/temperate genus *Protoparmelia* occurred much more recently, i.e. in the Miocene.

## Introduction

Tropical taxa are generally older than their extra-tropical relatives (Gaston and Blackburn 1996, Cattin et al. 2016). Age differences between tropical and extra-tropical species have been attributed to different climatic histories, environment and geography of the two regions (Dobzhansky 1950, Fischer 1960, Dubey and Shine 2011, Cattin et al. 2016, Richardson and Pennington 2016). Past glaciation events have mainly influenced extra-tropical regions, causing several waves of extinction (Weir and Schluter 2007, Rolland et al. 2014). Tropical regions did not experience the same climatic extremes faced by extra-tropical regions (Wallace 1878). Due to the stable climatic conditions and lower extinction rates, species have persisted longer in the tropical regions than the extra-tropical regions (Richardson and Pennington 2016). The ages of extant tropical and extra-tropical species have been well studied and compared for plants and animals (Moreau and Bell 2013, Kerkhoff et al. 2014, Cattin et al. 2016). However, there are only a few studies on the timing of diversification of closely related extant tropical and extra-tropical lichen-forming fungi (Kraichak et al. 2015, Lumbsch et al. 2008, Lumbsch 2016). Understanding the origins and diversification of tropical and extra-tropical taxa may be useful for explaining present patterns of species diversity and for identifying the mechanisms behind diversification.

Most lichenised fungi belong to the Lecanoromycetes within the Ascomycota. Within Lecanoromycetes, Parmeliaceae is the largest family of lichenised fungi consisting of approximately 2,500–3,000 species. This family has recently been divided into two subfamilies, Protoparmelioideae and Parmelioideae (Divakar et al. 2017, Kraichak et al. 2017). Although the diversification patterns of Parmelioideae and various clades within Parmelioideae are well-studied (Amo de Paz et al. 2011, Leavitt et al. 2012, Divakar et al. 2015), the diversification patterns of Protoparmelioideae remain unexplored. Interestingly, although closely related, the species diversity in the two subfamilies is drastically different, with Protoparmelioideae consisting of only about 25–30 species (Singh et al., 2015, Singh et al. 2017), in contrast to the species-rich Parmelioideae (Crespo et al. 2010, Thell et al. 2012). Unravelling the timing of the major diversification events of Protoparmelioideae may help understand the historical events that have led to the disparity in species richness of these two subfamilies. Furthermore, Protoparmelioideae consists of two genera inhabiting different climatic zones: the genus *Protoparmelia* consists predominantly of taxa inhabiting arctic and temperate regions, while the genus *Maronina* comprises mainly taxa inhabiting tropical and subtropical regions (Suppl. material 1; Kantvilas et al. 2010, Papong et al. 2011, Divakar et al. 2017). Protoparmelioideae therefore presents an opportunity to compare the divergence between closely-related species inhabiting tropical and extra-tropical regions.

Inferring the ancestral states of the characters, along with the diversification time, may help us understand how traits have evolved with respect to major geological events. For instance, the diversification of certain lineages in Parmelioideae may have been caused by key innovations that provided adaptive advantages, e.g. melanin production in *Melanohalea* (Divakar et al. 2013). Species richness of Parmelioideae has been linked to past climatic and geological events that provided new habitat and substrate opportunities (Amo de Paz et al. 2011, Leavitt et al. 2012, Kraichak et al. 2015). Inferring the ancestral habitat and substrate may provide useful insights into the diversity differences between the two sister clades.

The goals of this study were 1) to investigate whether tropical taxa have a different diversification history from extra-tropical taxa in Protoparmelioideae (Parmeliaceae) and 2) to infer the ancestral habitat and substrate in Protoparmelioideae to understand how these characters evolved within the subfamily.

## Materials and methods

### Dataset

We used the dataset from Singh et al. (2015) for estimating the divergence times in Protoparmelioideae (Suppl. material 2). This dataset is referred to as dataset 1 and it consists of 99 samples of *Protoparmelia* s. str. (11 species), 37 samples of the newly resurrected genus *Maronina* (12 species) and 73 taxa from close relatives of Protoparmelioideae, i.e. from Parmelioideae (40 taxa), Lecanoraceae (4 taxa), Gypsoplacaceae (2 taxa), Ramboldiaceae (10 taxa), *Protoparmelia* s. l. (24 taxa) and *Miriquidica* (12 taxa). This dataset comprises six loci: *RBP1* (696 bp), *TSR1* (756 bp), *MCM7* (655 bp), nuLSU (1064 bp), mtSSU (834 bp) and ITS (807 bp). Species concepts used in the current study are based on Singh et al. (2015). In short, this study inferred the independent evolutionary lineages in *Protoparmelia* and *Maronina* based on molecular data. Previously accepted taxa were considered putative species (12 described species). In addition, well-supported monophyletic clades in the six-locus concatenated ML and Bayesian phylogenetic trees (BS > 70%, PP > 0.95) were also considered as putative species, resulting in a 25-species-scenario. The marginal posterior probability of the 25-species-scenario was estimated using the programme BP&P v3, which utilises a reversible-jump Bayesian Markov chain Monte Carlo (MCMC) algorithm to infer the posterior probability of each delimited species and the posterior probability for the overall number of delimited species. The species tree from *BEAST was used to infer the speciation probabilities by BP&P (Yang and Rannala 2014). Further details of this analysis are mentioned in Singh et al. (2015).

Molecular dating can be done by using fossil records, substitution rates of genetic markers or by using the already estimated divergence date for a node in a phylogeny as the calibration point. The split of Protoparmelioideae from Parmelioideae has been shown to have occurred ~108 Ma (Amo de Paz et al. 2011) and 102 Ma (Kaasalainen et al. 2015, Divakar et al. 2017). We included the most recent estimate, i.e. by Divakar et al. (2017), to estimate the diversification times within Protoparmelioideae. We used a normal distribution (instead of a uniform prior), with the mean of 102.0 Ma and sdev = 9 Ma (Divakar et al. 2017) and truncated the upper and lower estimates of the split between Parmelioideae and Protoparmelioideae to 130 and 80 Ma, respectively. For each marker, we implemented the most appropriate model of DNA sequence evolution which was inferred using JModelTest (Darriba et al. 2012). We estimated divergence times in Protoparmelioideae by implementing a Birth-Death prior using an uncorrelated Bayesian relaxed molecular clock model (uncorrelated lognormal) and unlinked substitutions models across the loci as implemented in the programme BEAST v1.8.1 (Drummond et al. 2006, Drummond and Rambaut 2007). We performed the analysis with two independent Markov chain Monte Carlo (MCMC) runs of 50 million generations (10% burn-in), sampling one tree every 5000 generations (9000 trees obtained). We used the programme Tracer v1.6 to evaluate each chain and obtain the effective sample sizes for each parameter (Rambaut et al. 2014). Using TreeAnnotator version 1.8.0, the chains were combined to obtain the maximum clade credibility tree with mean node heights posterior distributions of estimated divergence dates (Drummond and Rambaut 2007).

### Identifying climatic zones of the clades

We extracted the climatic data for the *Protoparmelia* and *Maronina* species based on the coordinate information of the sampling sites. We used the global environmental stratification (GEnS) software, which is based on statistical clustering of bioclimatic data (Metzger et al. 2013). This is a high-resolution quantitative stratification of climatic data, which classifies the geographic regions of the world into 18 global environmental zones, based on a broad set of climate-related variables extracted from WorldClim (Hijmans et al. 2005, Metzger et al. 2013). The 18 global environmental zones are- A: arctic 1, B: arctic 2, C: extremely cold and wet 1, D: extremely cold and wet 2, E: cold and wet, F: extremely cold and mesic, G: cold and mesic, H: cool temperate and dry, I: cool temperate and xeric, J: cool temperate and moist, K: warm temperate and mesic, L: warm temperate and xeric, M: hot and mesic, N: hot and dry, O: hot and arid, P: extremely hot and arid, Q: extremely hot and xeric and R: extremely hot and moist. The 18 environmental zones are further grouped into seven broad biomes, namely arctic/alpine (environmental zones A, B, C & D), boreal/alpine (environmental zones E, F & G), cool temperate (environmental zones H, I & J), warm temperate (environmental zones K & L), subtropical (environmental zone M), dry lands (environmental zones N, O, P & Q) and tropical (environmental zone R; Metzger et al. 2013).

We performed linear discrimination analysis (LDA) using the package MASS in R (Venables and Ripley 2002) to infer if there is significant differentiation in the climatic variables between the *Protoparmelia* and *Maronina* inhabiting warm temperate regions. Linear discrimination analysis provides the linear combinations of the variables (here the 19 bioclimatic variables) that give the best possible separation between the groups i.e. in our study, taxa inhabiting warm temperate regions in *Protoparmelia* and *Maronina*. *Protoparmelia badia* B2 and *Maronina isidiata* E were excluded from this analysis as these species are represented by only two samples. We inferred the separation achieved by the discriminant function using the least correlated bioclim variables (first 4 bioclim variables) and calculated the mean values of the discriminant functions for each group.

### Ancestral state reconstruction

We reconstructed the ancestral habitat and substrate of Protoparmelioideae. We obtained information on habitat and substrate from literature (Aptroot et al. 1997, Nash et al. 2004, Lendemer and Lumbsch 2008, Coppins and Chambers 2009, Kantvilas et al. 2010, Papong et al. 2011). For the new species sensu Singh et al. (2015), information on habitat was inferred from the spatial framework analysis based on Metzger et al. (2013), which groups the global environment into seven “broad biomes” namely, arctic/alpine, boreal alpine, cool temperate, warm temperate, subtropical, dry lands and tropical. To infer the ancestral habitat of Protoparmelioideae, we grouped the “broad biomes” into cold (arctic/alpine, boreal alpine, cool temperate) and warm regions (warm temperate, subtropical, dry lands and tropical).

We used the 6-locus dataset from Singh et al. (2017), dataset 2, to infer the species tree using *BEAST as implemented in BEAST v2.2 (Drummond and Rambaut 2007). We used a Birth-Death process and gamma-distributed population sizes for the species tree prior and a pairwise linear population size model with a constant root. The closest model to the best-suggested model from jModelTest under the AICc criterion was selected as the substitution model for each locus (Suppl. material 3). Two independent Markov Chain Monte Carlo (MCMC) analyses were performed for a total of 50,000,000 generations, sampling every 1,000 steps. Convergence of the runs to the same posterior distribution and the adequacy of sampling (using the Effective Sample Size [ESS] diagnostic) were assessed with Tracer v1.6. The first 10% of the samples were removed as burn-in, resulting in 45,000 trees. 5,000 trees were then randomly sampled from these trees using the package ape in R, for inferring ancestral habitat and substrate and using BayesMultiState (Pagel and Meade 2006).

We reconstructed the ancestral habitat and substrate with binary character state coding using BayesMultiState implemented in BayesTraits version 3.0 (Pagel and Meade 2006). We employed the reversible-jump MCMC, where models are visited in proportion to their posterior probability. We traced the evolution of these characters on the molecular phylogeny using maximum likelihood (ML) and Bayesian inferences (BI) approaches. To account for phylogenetic mapping uncertainty, we evaluated probabilities of ancestral states calculated from the 5000 BI trees using the MCMC method in BayesMultiState, implemented in the BayesTraits v3.0. Ancestral states were then reconstructed for selected nodes for each character, which were selected based on their posterior probability support values of the BI analysis. We used a reversible-jump hyperprior with a gamma prior (exponential prior seeded from a uniform distribution on the interval 0 to 30) to reduce uncertainty of choosing priors in the MCMC analysis. Based on the preliminary analyses, we set the ratedev value to 8, to achieve an acceptance rate of proposed changes between 20 and 40% to ensure adequate mixing. The option “AddNode” was used to find the proportion of the likelihood associated with each of the possible states at each node. Three independent MCMC runs were performed with 2,000,000 iterations. Chains were sampled every 500^th^ iteration after a burn-in of 20,000 iterations (40 trees).

### Network analysis

Phylogenetically distant but geographically co-existing species may experience interspecies gene flow (Lumaret and Jabbour-Zahab 2009, Martin et al. 2013, Kol-Maimon et al. 2014). This might lead to the transfer of genes and the presence of similar characters in phylogenetically unrelated species (Makarenkov and Legendre 2004, Bapteste et al. 2013). Gene flow and homoplasy of characters can both be used to explain gain and loss of characters on a phylogenetic tree. We performed a network analysis to check if genetically distant species with similar characters are affected by interspecies gene flow.

We used PhyloNet to detect hybridisation events in the data while accounting for incomplete lineage sorting (Than et al. 2008). We applied the ML approach implemented in PhyloNet to infer the possibility of reticulation events in Protoparmelioideae, allowing up to two reticulations in 50 runs. The outgroup was excluded from the network analysis. We also ran multiple independent analyses on randomly selected subsets of 10 species, represented by one sample each due to the inability of PhyloNet to deal with the large dataset. The MCMC chain was run for 250,000 iterations and burn-in of 10,000 iterations (25 trees).

## Results

### Identifying climatic zones of the clades

We identified the habitat of different Protoparmelioideae taxa using GEnS (Suppl. material 4; Metzger et al. 2013). We found that, of the 11 *Protoparmelia* species, seven inhabit extremely cold to cold and cool temperate regions and four inhabit cool and warm temperate to warm temperate regions (Suppl. material 4). As for *Maronina*, eight species inhabit extremely hot to hot regions (zones M, N, Q and R according to Metzger et al. 2013) and four species inhabit warm temperate to warm temperate and hot regions (zones K, L, M, N and Q, Suppl. material 4). Linear discrimination analysis (LDA) showed that the mean values of the discriminant functions for each clade based on the most uncorrelated bioclim variables (first 4 bioclim variables, based on the scree plot; Suppl. material 5) was -3.517 and 1.034, respectively, for group 1 and group 2 and the misclassification rate was 4.54 (Suppl. material 6). The low rate of misclassification strongly supports the climatic difference between *Protoparmelia* and *Maronina* taxa inhabiting warm temperate regions. The stacked histogram clearly shows differentiation between the two groups (Suppl. material 5). Our results show that *Protoparmelia* and *Maronina* species inhabiting the broad “warm temperate biome” (Metzger et al. 2013) are well differentiated by more fine-scaled climate data.

### Divergence dating

**Figure 1.**
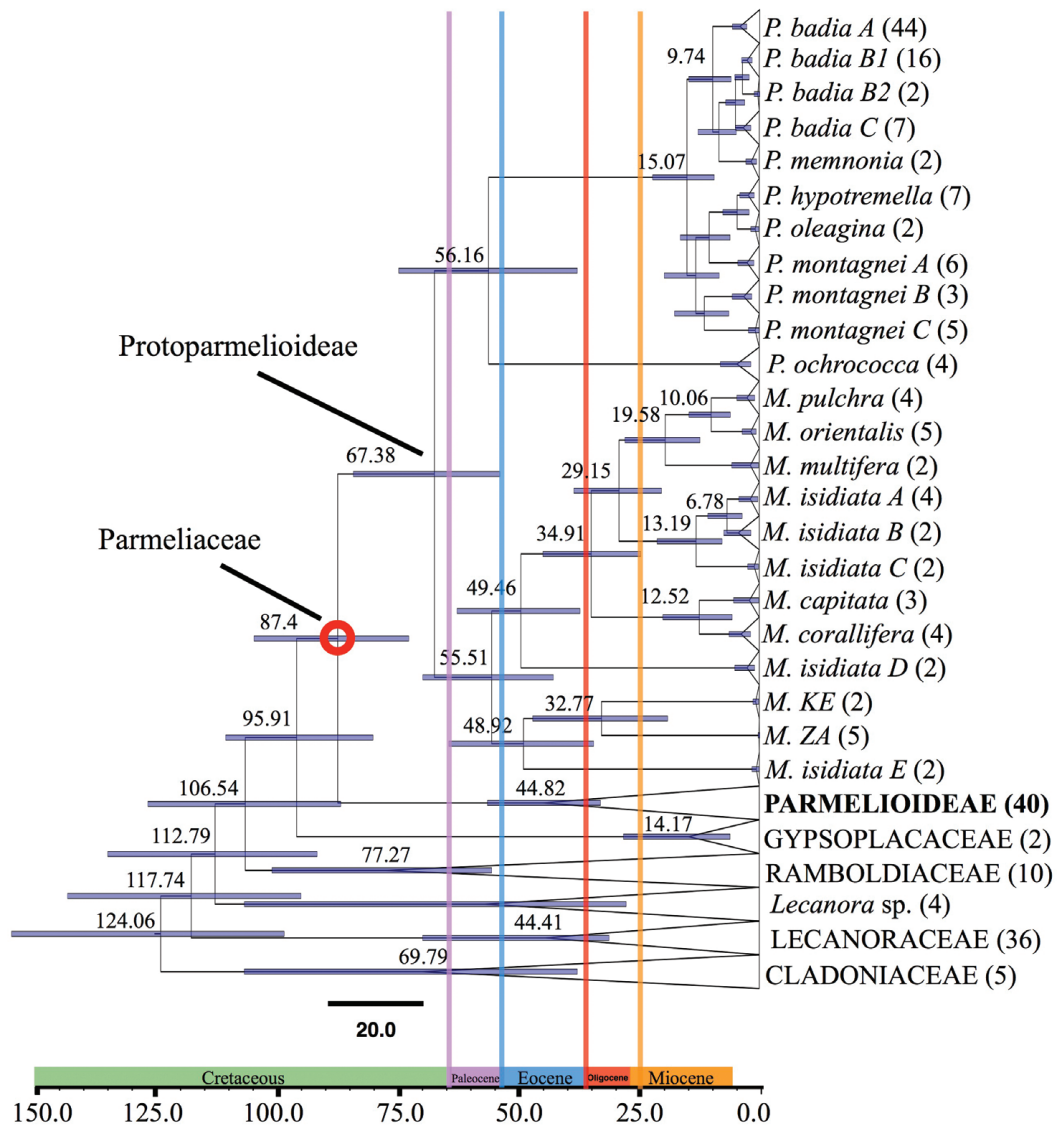
Time-calibrated phylogeny of the major lineages of Lecanorales (Lecanoraceae, Parmeliaceae, Ramboldiaceae, and Gypsoplacaceae), based on a six-locus dataset, dataset 1 (Singh et al. 2015). Cladoniaceae was used as outgroup (Arup et al. 2007, Singh et al. 2013). Mean node age, 95% highest posterior density (HPD) and posterior probability (PP) were mapped on the maximum clade credibility tree. The red circle indicates the calibration point, i.e. the split between Protoparmelioideae and Parmelioideae. Only the strongly supported nodes were considered for divergence time estimates. Geological times are indicated at the axis of the tree. The number of specimens per species is indicated in brackets in front of the taxon names. The scale at the bottom of the tree represents age in millions of years (Ma). Parmelioideae, Gypsoplacaceae, Cladoniaceae, Ramboldiaceae and Lecanoraceae clades are collapsed. In Parmelioideae, *Miriquidica* and *Protoparmelia* s. l. clades are collapsed at the species level.

The split between Parmelioideae and Protoparmelioideae occurred around 87 Ma during the Cretaceous. The tropical lowland genus *Maronina* split from the extra-tropical, arctic/temperate genus *Protoparmelia* around 67 Ma (Fig. 1, Table 1). Diversification within Protoparmelioideae occurred from the Oligocene to the early Pliocene. Most of the speciation events in both *Protoparmelia* and *Maronina* occurred during the Miocene (Table 1).

**Table 1.** The dates of origin of lineages in Protoparmelioideae and the initial divergence of Protoparmelioideae from Parmelioideae (ancestral splits).

Lineage	Mean	Range (95% credibility intervals)
Origin of Ramboldiaceae	106.54	95% HPD = 86.77–126.7
Origin of Gypsoplacaeae	95.91	95% HPD = 80.09–110.59
Parmelioideae-Protoparmelioideae split	87.4	95% HPD = 72.68–104.72
*Protoparmelia-Maronina* split	67.38	95% HPD = 53.78–84.16
Origin of *Protoparmelia ochrococca*	56.16	95% HPD = 37.8–74.75
*Protoparmelia badia A*	9.74	95% HPD = 5.94–14.69
*Protoparmelia memnonia*	8.45	95% HPD = 4.86–12.76
*Protoparmelia badia C*	5.05	95% HPD = 1.86–5.03
*Protoparmelia badia B1*	3.57	95% HPD = 2.19–5.17
*Protoparmelia badia B2*	3.57	95% HPD = 2.19–5.17
*Protoparmelia oleagina*	11.47	95% HPD = 6.42–17.63
*Protoparmelia hypotremella*	11.47	95% HPD = 6.42–17.63
*Protoparmeliamontagnei A*	4.68	95% HPD = 2.2–7.64
*Protoparmeliamontagnei B*	4.68	95% HPD = 2.2–7.64
*Protoparmeliamontagnei C*	10.47	95% HPD = 6.15–16.43
*Maronina pulchra*	10.06	95% HPD = 6.09–14.66
*Maronina orientalis*	10.06	95% HPD = 6.09–14.66
*Maronina multifera*	19.58	95% HPD = 12.39–27.91
*Maronina isidiata A*	6.78	95% HPD = 3.65–10.76
*Maronina isidiata B*	6.78	95% HPD = 3.65–10.76
*Maronina isidiata C*	13.19	95% HPD = 7.8–21.26
*Maronina capitata*	12.52	95% HPD = 5.72–20.07
*Maronina corallifera*	12.52	95% HPD = 5.72–20.07
*Maronina isidiata D*	49.46	95% HPD = 37.23–62.68
*Maronina isidiata E*	48.92	95% HPD = 34.4–64.39
*Maronina ZA*	32.77	95% HPD = 19.08–47.02
*Maronina KE*	32.77	95% HPD = 19.08–47.02

**Figure 2.**
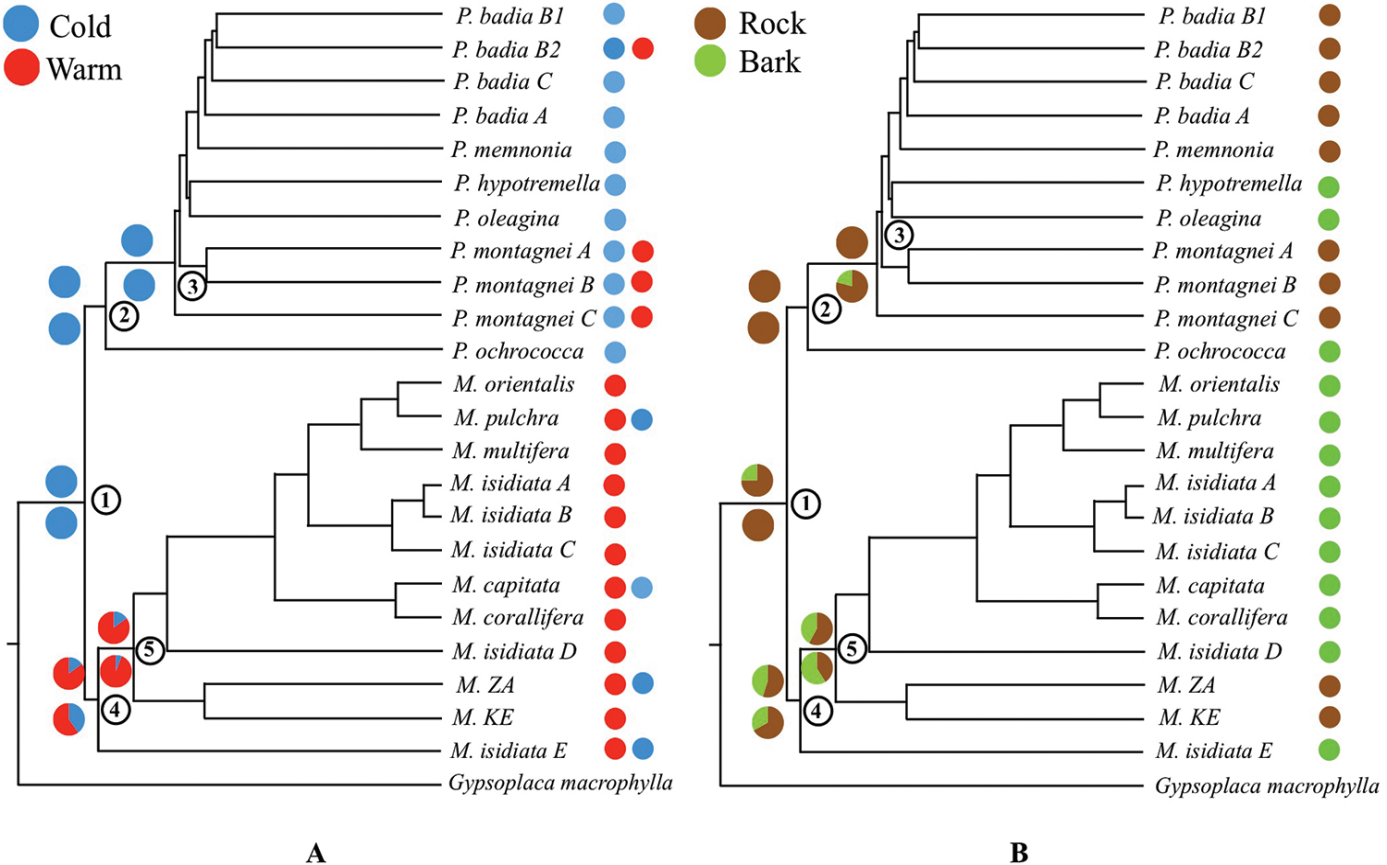
Ancestral states in Protoparmelioideae: Chronogram based on a six-locus dataset, dataset 2 (Singh et al. 2017), representing a species tree of Protoparmelioideae showing the ancestral states at nodes of interest. The topology is derived from the *BEAST species tree. A consensus tree was generated in TreeAnnotator. The current substrate of each species is indicated by the coloured circles in front of the name of the species. Polymorphic taxa have more than one coloured circle. Nodes at which ancestral states are reconstructed are numbered from 1 to 5. Pie charts indicate probabilities of each ancestor being in each of the two potential states at nodes of interest. The circles above the node represent bootstrap support for each character state and the circles at the bottom represent the posterior probability. A) Ancestral habitat: cold (blue), warm (red) and B) Ancestral substrate: rock (brown), bark (green).

### Ancestral state reconstruction

We reconstructed the ancestral habitat and substrate of Protoparmelioideae using ML and Bayesian approaches. We did not find any conflict between the two approaches and both approaches supported a similar character at the investigated nodes. The Bayesian analysis was run three times for each character at each node and we did not find any conflict amongst the three runs (Table 2). We found that the ancestor of Protoparmelioideae was a rock-dwelling and cold environment inhabiting lichen-forming fungus (Fig. 2).

**Table 2.** Results of the character reconstruction for Protoparmelioideae using MCMC and ML methods. We report the posterior probabilities (PP) and likelihoods for the ancestral habitat and substrate at five nodes from Fig. 2. Values with bootstrap support >0.70 and PP >0.95 are marked in bold.

**Node**	**Approach**	**Habitat**		**Substrate**	
		**(P) cold**	**(P) warm**	**(P) rock**	**(P) bark**
1	ML	**1.000**	0.000	**0.755**	0.250
	Bayesian	**1.000**	0.000	**1.000**	0.000
2	ML	**0.900**	0.099	**0.780**	0.220
	Bayesian	**1.000**	0.000	0.578	0.422
3	ML	**1.000**	0.000	**0.995**	0.005
	Bayesian	0.060	0.940	0.880	0.120
4	ML	**1.000**	0.000	**0.756**	0.244
	Bayesian	**1.000**	0.000	0.756	0.234
5	ML	0.188	**0.812**	**0.551**	0.449
	Bayesian	0.048	**0.952**	0.700	0.300

### Network analysis

Network analysis was performed to infer events such as hybridisation and gene flow in Protoparmelioideae. Our analysis indicates that reticulation events are unlikely amongst species in Protoparmelioideae. We did not find any cases of hybridisation amongst taxa in Protoparmelioideae.

## Discussion

In this study, we investigated the diversification timing in Protoparmelioideae. The sister-relation between Protoparmelioideae and Parmelioideae was supported in our analysis as in previous studies (Arup et al. 2007, Singh et al. 2013, Divakar et al. 2015, Divakar et al. 2017). Protoparmelioideae comprises two genera, *Protoparmelia*, which includes taxa with predominantly extra-tropical distribution and *Maronina*, which mainly comprises species with tropical distribution (Divakar et al. 2017). We found that *Protoparmelia* split from *Maronina* around 67 Ma. Our analysis suggests that clade diversification events in *Protoparmelia* and *Maronina* occurred at different geological time scales.

### Are tropical taxa older?

Our study suggests that clade diversification events within *Maronina* predate those in *Protoparmelia*. These results are in line with the hypothesis that tropical taxa are older than their arctic/temperate relatives (Dobzhansky 1950, Mittelbach et al. 2007, Schemske 2009). One reason for this is the different climatic history of these regions. Due to major climatic perturbations, the arctic/temperate regions may have suffered waves of extinction. On the contrary, subtropical/tropical regions had a comparatively stable climate and escaped major glaciation events and, thus, did not face major extinctions (Willig et al. 2003, Wiens and Donoghue 2004, Mittelbach et al. 2007). Although the tropics escaped glaciation, these regions did face climatic perturbations in the form of severe aridification that impacted species’ ranges and also led to extinctions and populations bottlenecks (Demenou et al. 2016, Powell and Glazier 2017). This could explain the comparable species diversity between *Protoparmelia* and *Maronina*, as well as the restricted ranges of *Maronina* species. *Maronina* species, which were thought to have a broad geographic distribution, i.e. *M. isidiata*, have been shown to comprise five distinct lineages/species (Singh et al. 2015). On the contrary, *Protoparmelia badia*, *P. hypotremella* and *P. oleagina* have a broad geographic distribution. A recent study suggested that *P. badia* and *P. montagnei* comprise different morphospecies, however, one lineage of *P. badia* is cosmopolitan and has a broad geographic distribution (Singh et al. 2015). The other putative lineages in *P. badia* and *P. montagnei* are recently discovered and, so far, they have been reported only from Spain and Italy.

### Diversification patterns

The diversification of *Protoparmelia* involves an initial “lag phase”, indicated by a clade with a long branch (spanning ~50 million years in *Protoparmelia*). However, a long branch might be caused by several factors including extinction of taxa, founder effects or artefacts of the dataset (incomplete sampling etc.). Incomplete sampling might not be the case for the observed long branch in *Protoparmelia* as molecular data is available for most of the taxa and only the taxa forming a monophyletic clade as *Protoparmelia* s. str. (sensu Singh et al. 2013, 2015) were included in this study. These studies showed *Protoparmelia* to be polyphyletic and many taxa have been moved to *Ramboldia (P. plicatula*, *P. petraeoides*), *Maronina* or *Lecanora* (*P. ryaniana*).

Considering the climatic history of the arctic/temperate regions where *Protoparmelia* species are predominantly distributed, extinction could be assumed as the one of the main reasons resulting in the observed long branch in *Protoparmelia*. On the other hand, under comparatively stable climatic conditions, little or no extinction of the early diverging branches might have led to the more even branching pattern in *Maronina*. Thus, past climate, geographic position and geological events might have caused differences in the timing of speciation events between *Protoparmelia* and *Maronina*.

### Phylogenetic network

Evolution of organisms is often represented by a phylogenetic tree, which assumes vertical transfer of genetic material from ancestors to descendants. Evolutionary relationships however, might be more complicated and genes may be transferred horizontally between different or reproductively isolated organisms (Lumaret and Jabbour-Zahab 2009, Kol-Maimon et al. 2014). Sharing of genetic material between species may lead to shared characters despite their phylogenetic related nature (Makarenkov and Legendre 2004, Bapteste et al. 2013). In Protoparmelioideae, *Protoparmelia* is predominantly saxicolous (8 species) with only three corticolous species whereas *Maronina* is predominantly corticolous (10 species) with only two saxicolous species. We inferred whether or not the similar substrates or habitat preference in phylogenetically distant species might be due to the gene flow between them. Our analysis suggests that hybridisation events are unlikely to have occurred between taxa in Protoparmelioideae and the similar substrate and habitat preference between *Protoparmelia* and *Maronina* are probably results of independent evolution of characters.

### Ancestral habitat and substrate of Protoparmelioideae

Our results suggest that the ancestors of Protoparmelioideae as well as *Protoparmelia* probably inhabited cold environments (Fig. 2). *Protoparmelia* split from *Maronina* ~67 Ma ago (Fig. 1) and subsequently diversified in arctic/temperate regions in the Northern hemisphere. The cold inhabiting ancestors might have facilitated the diversification in the arctic/temperate regions when presented with novel geographical and ecological opportunities due to mountain uplifts.

Substrate is an important factor determining lichen distribution. For instance, major diversification events within the epiphyte-rich subclasses within Ascomycota occurred in the Jurassic and Cretaceous (Prieto and Wedin 2013), the latter being the period of origin and diversification of angiosperms. In our study, we found evidence that the ancestor of Protoparmelioideae was rock dwelling (Fig. 2). This is connected to the fact that the ancestor of Protoparmelioideae was also inhabiting cold, vegetation-poor, habitats. Substrates other than rock were not available.

## Supplementary materials

Supplementary material 1 Distribution of *Protoparmelia* and *Maronina* species. This is an open access article distributed under the terms of the Creative Commons Attribution License (CC BY 4.0), which permits unrestricted use, distribution, and reproduction in any medium, provided the original author and source are credited.Garima Singh, Francesco Dal Grande, Jan Schnitzler, Markus Pfenninger, Imke Schmitt

Supplementary material 2 Voucher information This is an open access article distributed under the terms of the Creative Commons Attribution License (CC BY 4.0), which permits unrestricted use, distribution, and reproduction in any medium, provided the original author and source are credited.Garima Singh, Francesco Dal Grande, Jan Schnitzler, Markus Pfenninger, Imke Schmitt

Supplementary material 3 Genetic characteristics of nuclear loci used in this study This is an open access article distributed under the terms of the Creative Commons Attribution License (CC BY 4.0), which permits unrestricted use, distribution, and reproduction in any medium, provided the original author and source are credited.Garima Singh, Francesco Dal Grande, Jan Schnitzler, Markus Pfenninger, Imke Schmitt

Supplementary material 4 Global environmental zones of the *Protoparmelia* and *Maronina* species This is an open access article distributed under the terms of the Creative Commons Attribution License (CC BY 4.0), which permits unrestricted use, distribution, and reproduction in any medium, provided the original author and source are credited.Garima Singh, Francesco Dal Grande, Jan Schnitzler, Markus Pfenninger, Imke Schmitt

Supplementary material 5 Results of the linear discrimination analysis This is an open access article distributed under the terms of the Creative Commons Attribution License (CC BY 4.0), which permits unrestricted use, distribution, and reproduction in any medium, provided the original author and source are credited.Garima Singh, Francesco Dal Grande, Jan Schnitzler, Markus Pfenninger, Imke Schmitt

Supplementary material 6 Results of the linear discrimination analysis This is an open access article distributed under the terms of the Creative Commons Attribution License (CC BY 4.0), which permits unrestricted use, distribution, and reproduction in any medium, provided the original author and source are credited.Garima Singh, Francesco Dal Grande, Jan Schnitzler, Markus Pfenninger, Imke Schmitt
