# Supplementary material for: Different diversification histories in tropical and temperate lineages in the ascomycete subfamily Protoparmelioideae (Parmeliaceae)
Source: MycoKeys. 2018 Jul 2;(36):1–19. doi: 10.3897/mycokeys.36.22548 (PMC6037653; doi:10.3897/mycokeys.36.22548)
Supplement: Supplementary material 1 — Distribution of Protoparmelia and Maronina species. [file mycokeys-36-001-s006.pdf]

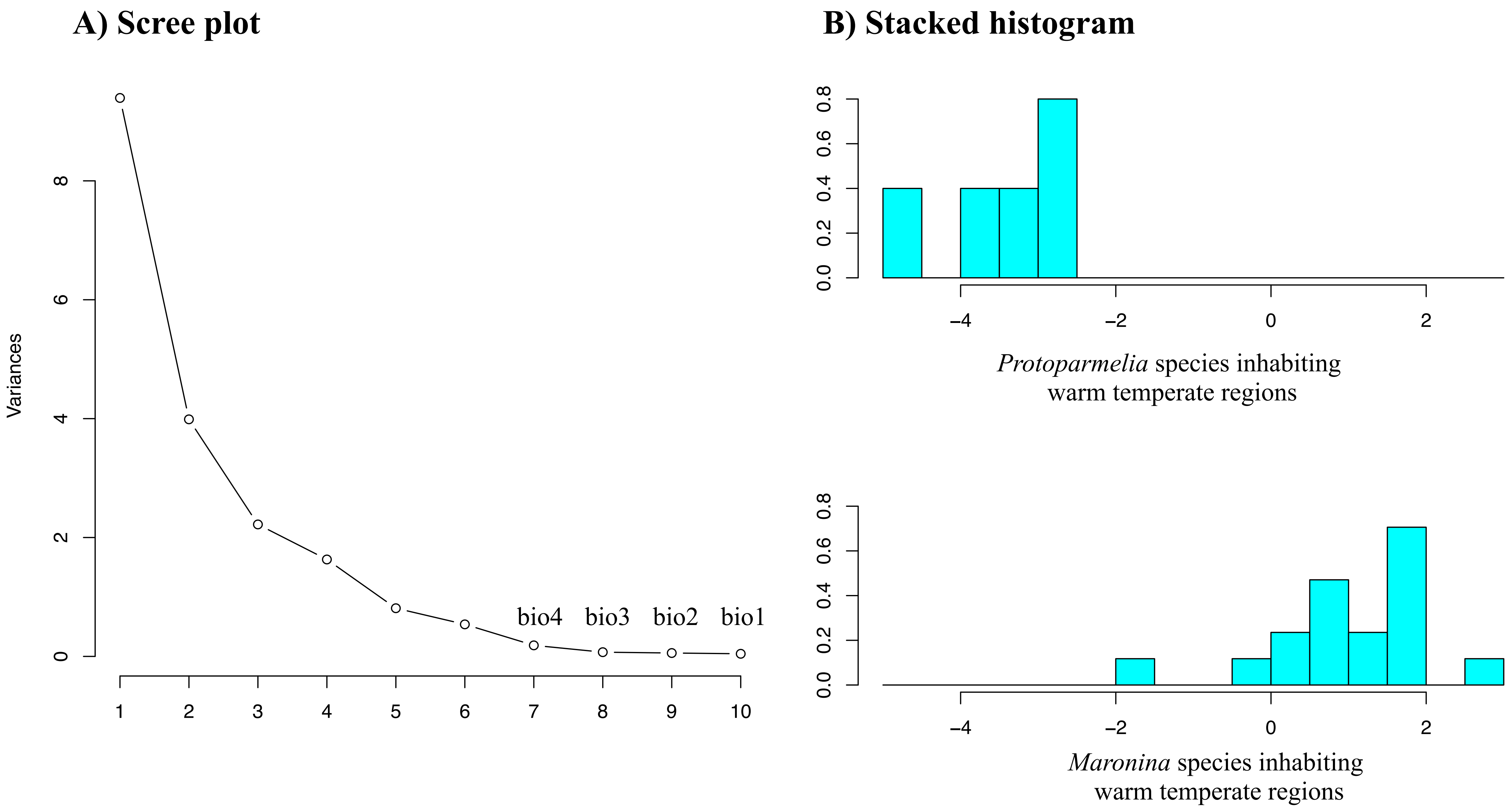

Linear discrimination analysis **A)** scree plot summarising the results of principal components analysis for deciding the principal components to retain. The change in slope or the elbow of scree plot occurs at component number 4,, i.e., bio4 which is the bioclimatic variable. The four bioclimatic variables stand for, bio1 = Annual Mean Temperature, bio2 = Mean Diurnal Range (Mean of monthly (max temp - min temp)), bio3 = Isothermality (BIO2/BIO7) (* 100) and bio4 = Temperature Seasonality (standard deviation *100); **B)** Stacked histogram of the values of the discriminant function for *Protoparmelia* and *Maronina* species inhabiting warm temperate regions. The samples of the two genera are well separated by the first discriminant function, and there is no overlap in values.
